# Low Salinity Improves Photosynthetic Performance in *Panicum antidotale* Under Drought Stress

**DOI:** 10.3389/fpls.2020.00481

**Published:** 2020-05-29

**Authors:** Tabassum Hussain, Hans-Werner Koyro, Wensheng Zhang, Xiaotong Liu, Bilquees Gul, Xiaojing Liu

**Affiliations:** ^1^Center for Agricultural Resources Research, Institute of Genetics and Developmental Biology, Chinese Academy of Sciences, Shijiazhuang, China; ^2^Dr. Muhammad Ajmal Khan Institute of Sustainable Halophyte Utilization, University of Karachi, Karachi, Pakistan; ^3^Institute of Plant Ecology, Justus Liebig University Giessen, Giessen, Germany

**Keywords:** water deficit, salt resistance, combined stress, photosynthesis, halophyte, stomatal and biochemical limitations

## Abstract

Salinity and drought are two often simultaneously occurring abiotic stresses that limit the production of food crops worldwide. This study aimed to distinguish between the separate and combined impacts of drought and salinity on the plant response. *Panicum antidotale* was cultivated in a greenhouse under the following growth conditions: control, 100 mM NaCl (100) and 300 mM NaCl (300) salinity, drought (D; 30% irrigation), and two combinations of salinity and drought (100 + D and 300 + D). The growth response was as follows: 0 ≈ 100 > 100 + D > > D ≈ 300 ≈ 300 + D. Growth correlated directly with photosynthesis. The net photosynthesis, stomatal conductance, intercellular CO_2_, transpiration, ribulose 1,5-bisphosphate carboxylase (Rubisco), ribulose 1,5-bisphosphate (RuBP) regeneration, and triose phosphate utilization protein (e.g., phosphoenolpyruvate carboxylase) were highest in the control and declined most at 300 + D, while 100 + D performed significantly better as compared to drought. Maximum and actual photosystem II (PSII) efficiencies, along with photochemical quenching during light harvesting, resemble the plant growth and contemporary CO_2_/H_2_O gas exchange parameters in the given treatments. Plant improves water use efficiency under salt and drought treatments, which reflects the high water conservation ability of *Panicum*. Our findings indicate that the combination of low salinity with drought was able to minimize the deleterious effects of drought alone on growth, chlorophyll content, cell integrity, photosynthesis, leaf water potential, and water deficit. This synergetic effect demonstrates the positive role of Na^+^ and Cl^–^ in carbon assimilation and osmotic adjustment. In contrast, the combination of high salinity and drought enforced the negative response of plants in comparison to single stress, demonstrating the antagonistic impact of water availability and ion toxicity.

## Introduction

The rapid change in global climate threatens plant growth and productivity worldwide. These changes are seriously abrupt and can be present in combinations at any given time (Qadir et al., 2014). Alterations in abiotic factors like water, salinity, temperature, soil composition, irradiance, ultraviolet radiations, nutrients, flooding, etc., are some of the examples of such changes. Tremendous efforts had been made to address the impacts of each of these abiotic stressors on plant growth (Argus et al., 2015; Hatfield and Prueger, 2015; Liu et al., 2017; Negrao et al., 2017). However, the number of studies to unravel the combined effect of multiple stress factors on plants’ productivity are very few (Mittler, 2006; Zandalinas et al., 2018). The combination of the different stresses causes either a positive (synergetic) or negative (antagonistic) influence on plant performance when studied at the levels of morphology, biochemistry, and physiology (see the review of Zandalinas et al., 2018 and other references within). The interaction of water deficit (drought) and salinity is a common co-occurring constraint (Hu and Schmidhalter, 2005) that has been paid less attention to. The increase in dry lands is often linked to inappropriate irrigation with saline water resources. Thus, a progressive and consecutive development of drought and salinity stress decreases the availability of cultivable land (Abdelraheem et al., 2019). This alarming situation should be resolved at the earliest. For this, a detailed understanding of the interactions of both stressors on plant growth needs to be undertaken.

Soil salinity imposes ionic (ion toxicity and imbalance) and osmotic stress (water deficiency) by lowering the soil water potential. Low values of soil matric potential (due to excessive salts) also cause water deficit, a primary physiological constraint for plant growth (Duarte et al., 2013). However, the contribution of these factors varies according to the type of soil. Excessive Na^+^ and Cl^–^ hinder vital physiological and biochemical mechanisms (e.g., CO_2_ assimilation). Halophytes are defined as species adapted to perpetually saline conditions. Salinity can even stimulate their growth by the accumulation of Na^+^ and Cl^–^, i.e., cheap osmotica (Cheeseman, 2015). Such physiological mechanisms are well established in the literature (Muchate et al., 2016). In contrast, there exist a few studies on the combined effect of salinity and drought, and that, too, are on glycophytes, the plants of a non-saline environment, where reduction in plant biomass is a common finding (Ahmed et al., 2013). Xero-halophytes, the plants capable of thriving both salinity and drought stress, seem a more promising focus for such studies. Gaining such knowledge will assist in their utilization for phytoremediation and in the development of xero-halophytic crops, i.e., crops with improved tolerance to drought and salinity stresses. The latter task can be achieved by following a simple breeding procedure between a crop species and a related stress-tolerant species (Cheeseman, 2015). This is a long-desired goal considered in the context of problems such as increasing desertification, salinization of agricultural land, increasing misuse of saline irrigation, and decreasing acreage (Abdelraheem et al., 2019).

Plant biomass production and net photosynthesis are correlated (Flexas et al., 2012b). The latter is a non-invasive indicator of non-optimal environmental conditions for plant growth. Net photosynthesis rate, under stress conditions, decreases due to stomatal and non-stomatal limitations, such as ribulose 1,5-bisphosphate carboxylase (Rubisco) enzyme activity, regeneration of ribulose 1,5-bisphosphate (RuBP), triose phosphate utilization, electron transport rate, the efficiency of photosystem II (PSII), etc. (Chaves et al., 2009; Chen et al., 2015). A large number of studies had been conducted to elucidate photosynthesis-limiting steps during stress conditions (such as salinity and drought) (Chen et al., 2015; Wang et al., 2017; Bellasio et al., 2018).

Stomatal closure appears as a first major response due to salinity (osmotic effect) that ultimately limits CO_2_ assimilation. Nevertheless, this is not the case for every halophyte as the efficiency of PSII (*F*_v_/*F*_m_ and ΦPS2), electron transportation rate (ETR), and other biochemical factors also play a role in limiting photosynthesis (Wang et al., 2017). Accumulated Na^+^ and Cl^–^ directly inhibit the activity of the Rubisco enzyme (Koyro et al., 2013; Galmés et al., 2017). The maximum and actual quantum efficiency of PSII was decreased in some plant species, while others are capable of resisting this reduction under stress (Koyro et al., 2013; Asrar et al., 2017). Reduction in ETR slows down the photochemical reactions, but it also protects the cell from electron leakage due to the damage in photosynthetic pigments (Tezara et al., 1999). Likewise, non-photochemical quenching (NPQ) enhances the photoprotective mechanism when a plant cannot properly channel the incident light energy to photochemistry. Thus, this competition between NPQ and photochemistry lowers the use of available light energy into photosynthesis. NPQ benefits plants when a decrease in gaseous exchange (due to closed stomata) reduces carbon fixation (Calvin cycle) and light energy becomes excessive to its utilization in light reactions. The rate-limiting factors of photosynthesis under salinity have been discussed in detail. A very few studies have focused on water deficit experiments while quantifying these limiting factors (Tezara et al., 1999; Galmés et al., 2017).

The closure of the stomata limits carbon uptake; however, it reduces the loss of water. Thus, plants maintain tissue osmotic conditions under stress (i.e., salt and drought). Such advantage becomes more apparent in the case of plants with C_4_ (i.e., an efficient) carbon fixation mechanism. These plants achieve high water use efficiency and are competitively better than plants possessing the C_3_ mechanism, a common carbon fixation pathway. In this study, we hypothesized whether the combination of salinity with a water deficit condition in the C_4_ plant *Panicum antidotale* minimizes the impact of stress due to its advantageous C-fixing mechanism and high water use efficiency. The reason for selecting this test species is its wide and natural distribution in plant-deprived lands (e.g., water deficit, saline, high temperature, etc.). Also, it is often regarded for its potential as an alternate crop (Ashraf, 2003; Ahmad et al., 2009; Khan et al., 2009; Hussain et al., 2015). These observations provoked our interest to determine its adaptation mechanisms in response to a combination of stress factors as the combined effect of salt and drought stress on the photosynthetic capacity of plants has not been investigated earlier, to the best of our knowledge. We designed this study to quantify the photosynthetic responses of *P. antidotale* in response to the combined stress of salinity and water deficit, i.e., conditions comparable to its natural environment. The specific objectives of the studies were to: (1) correlate photosynthetic responses with growth and water relations and (2) distinguish the stomatal and non-stomatal limitations of photosynthesis.

## Materials and Methods

### Plant Culture and Stress Treatments

Seeds were collected from natural mono-stand populations from the coast of Karachi, Pakistan, and were surface sterilized with 1% sodium hypochlorite for 1 min. Seeds were allowed to germinate in a growth chamber (25°C/14°C and 14/10-h day/night regimes) in perlite. Equal-sized three-leaf stage seedlings were transplanted to a greenhouse (28°C/16°C ± 2 and 14/10 h day/night regimes, 40–60% humidity, 600 ± 45 μmol photon m^–2^ s^–1^ light) in pots (15 × 22 cm, three plants/pot and three pots for each treatment) containing about 4 kg quartz sand (1–2 mm diameter) and irrigated with half-strength Hoagland’s nutrient solution (Epstein, 1972). Stress treatments were introduced after 2 weeks of seedling establishment. Plants were subjected to the following treatments: control (CK), salinity with 100 and 300 mM NaCl (S), drought (D), and a combination of salinity and drought (S + D). The drought-treated pots were irrigated 30% (by volume) of solution as compared to the control pots (i.e., irrigated with maximum field capacity), while in the case of the S + D treatment, 100 mM + nutrient solution (100 + D) and 300 mM + nutrient solution (300 + D) with the same volume as in the drought treatment. The salinity and drought treatments were introduced in steps: 50 mM NaCl in the morning and evening, and drought was achieved after 4–5 days in all drought treatments. The NaCl concentrations and drought conditions were maintained daily by a gravimetric approach and nutrient solutions were replaced every 3 days to avoid nutrient deficiencies. The pots’ positions were replaced randomly in the greenhouse to minimize block effects.

### Plant Biomass

Three plants from three pots were harvested after 4 weeks of stress treatments, and fresh weight was recorded immediately while dry weight was measured after drying at 70°C in an oven until a constant weight was achieved (>48 h).

### Water Relations

Leaf water content (WC) was calculated by subtracting the leaf dry weight from the fresh weight and expressing it as percent of fresh weight. The relative water content (RWC) was estimated by the method of Sharp et al. (1990). Leaf discs (more than eight) of 1 cm diameter (avoiding the margins and midrib) were cut and fresh weight (FW) was recorded. Five milliliters of deionized water was poured on these discs overnight at 4°C and then turgid weight (TW) was determined. Leaf discs were dried at 65°C for ∼48 h and, subsequently, dry weight (DW) was measured and the relative water content [RWC = (FW − DW)/(TW − DW) × 100] was calculated. Leaf water potential was determined at predawn by a dew point potential meter (WP4 dew point potentiometer, Decagon Devices, Pullman, NE, United States). Therefore, leaf samples were cut into sample pieces (∼1–2 mm diameter discs) to cover the 40 mm cup of the WP4 dew point potentiometer. The water potential (ψ_w_) was expressed in megapascals.

### Photosynthesis Measurements

Gas exchange and chlorophyll fluorescence measurements were performed on the first fully expanded (third and fourth) leaf by using an infrared gas analyzer, IRGA (LI6400XT, LI-COR Biosciences, Lincoln, NE, United States), equipped with red–blue LED chamber (2 cm^2^ area; 6400-40, LI-COR Biosciences). The light response curve (*P*_n_–PAR, photosynthetic active radiation), CO_2_ response curve (*P*_n_–*C*_i_), and chlorophyll fluorescence were measured on three different plants (each from a different pot) on each treatment. All measurements were performed from 0830 to 1500 h.

### Light Response Curves

Photosynthesis light response curves (*P*_n_–PAR) were measured after leaf acclimation at 2,000 μmol photon m^–2^ s^–1^ for about 40–50 min to stabilize stomatal conductance at *C*_a__tm_ 400 μmol CO_2_ m^–2^ s^–1^ with a flow rate of 300 μmol s^–1^ and humidity was about 40–65%. A stepwise decrease of the photosynthetic photon flux density (PPFD) from 2,000 to 0 μmol photon m^–2^ s^–1^ was carried. Measurements were taken at each light step after about 7–10 min, at a steady and stabilized response of the leaf. In accordance with Schulte et al. (2003), a non-linear exponential function was applied to the light response curve.

(1) f⁢(x)=a-exp⁡[b⁢(-x)]⁢c

where *f*(*x*) is the net carbon assimilation and *x* the incident PPFD on leaf. The coefficients (*a*), (*b*), and (*c*) were calculated by Newton’s least-square method. Coefficient (*a*) represents the maximum carbon flux at the saturated light, while dark respiration (*R*_d_) was estimated as the [(*a*) − (*c*)]. The saturation irradiance (*I*_s_), light compensation point (*I*_c_), and apparent quantum yield of CO_2_ assimilation (Φ_CO__2_) were calculated using the following equations:

(2) $$I_{\text{s}}=\ln\left(0.1.\frac{a}{c}\right).\left(-\frac{1}{b}\right)$$

(3) $$I_{\text{c}}=\ln\left(\frac{a}{c}\right).\left(-\frac{1}{b}\right)$$

(4) $$\Phi_{\mathrm{CO2}}=\exp\exp\left[b\,\left(-\text{Ic}\right)\right]\,c\,b$$

At saturated PPFD, the net photosynthesis rate (*P*_n_), stomatal conductance (*g*_s_), intercellular CO_2_ (*C*_i_), transpiration (*E*), intrinsic water use efficiency (WUEi: *P*_n_/*g*_s_), ETR/*P*_ngross_ (*P*_gross_ = *P*_n_ + dark respiration), and *C*_i_/*C*_atm_ were also recorded at saturated PPFD of three different plants in each treatment.

#### Photosynthesis CO_2_ Response Curves

The CO_2_ response curves were measured at light saturation at 1,500 μmol photon m^–2^ s^–1^ PPFD at a temperature of 28°C, 60% humidity, 1.0–1.5 kPa vapor pressure deficit (VPD), and 300 μmol s^–1^ flow rate by the LED chamber (2 cm^2^ area; 6400-40, LI-COR Biosciences). Leaves were acclimated for 40–50 min to achieve a constant net photosynthesis and stomatal conductance at a *C*_a__tm_ of 400 μmol CO_2_ m^–2^ s^–1^ before the induction of the CO_2_ response curve. A series of *C*_atm_ ranging from 1,500 to 0 μmol CO_2_ m^–2^ s^–1^ (400, 300, 200, 100, 50, 0, 400, 400, 600, 1,000, and 1,500) was adjusted until a stable reading of the net photosynthesis, intercellular CO_2_, and stomatal conductance (about 7 min at each change) for constructing the photosynthesis CO_2_ response curves on the three different plants of each treatment. According to Von Caemmerer (2000), we estimated Rubisco carboxylase activity (*V*_cmax_) and RuBP regeneration in terms of *J*_max_.

#### Chlorophyll Fluorescence and Chlorophyll Content

Chlorophyll *a* fluorescence was measured simultaneously with the *P*_n_–*C*_i_ response curves by using the same LED chamber (2 cm^2^ area; 6400-40, LI-COR Biosciences). About 30-min dark-adapted leaves were used for the minimal fluorescence (*F*_o_) at 0.5 μmol photon m^–2^ s^–1^, while the maximum fluorescence (*F*_m_) was recorded after applying a light saturating pulse (>8,000 μmol photons m^–2^ s^–1^ for 0.8 s). The maximum quantum efficiency of PSII reaction centers (*F*_v_/*F*_m_ = *F*_m_ − *F*_o_/*F*_m_) was calculated (Kitajima and Butler, 1975). The leaf was adapted to saturated light for about 40–50 min and then steady-state fluorescence (*F*_s_) and the maximum quantum efficiency of PSII of the light-adapted leaf (*F*’_m_) were recorded. The quantum efficiency of PSII (ΦPS2), non-photochemical quenching (NPQ), and photochemical quenching (qP) were determined using the following equations (Genty et al., 1989; Kooten and Snel, 1990):

(5) $$\Phi \,\text{PS}\,2=\frac{F_{\text{m}}^{\prime }-F_{\text{s}}}{F_{\text{m}}^{\prime }}$$

(6) $$\text{NPQ}=\frac{F_{\text{m}}}{F_{\text{m}}^{\prime }}-1$$

(7) $$\text{qP}=\frac{F_{\text{m}}^{\prime }-F_{\text{s}}}{F_{\text{m}}^{\prime }-F_{\text{o}}^{\prime }}$$

ΦPS2 represents the electron transport in PSII of the photosynthesis per absorbed photon. The electron transport rate (ETR) was also calculated as:

(8) ETR=Φ⁢PS⁢2.PPFD⁢.0.5.0.87

where 0.5 is the assumption of equal distribution of incident light between PSI and PSII and 0.87 indicates the leaf light absorbance.

The chlorophyll content of leaves was measured with a SPAD 502 chlorophyll meter without causing damage to plants on the same leaf, which has been selected for gas exchange measurements. A minimum of 15 readings were performed on one leaf and the data expressed as the mean of three different plant leaves.

#### Photosynthetic Proteins

Fully matured leaves were taken from the plant and immediately frozen in liquid nitrogen. About 0.1 g of ground leaves was vortexed in an extraction buffer (125 mM Tris–HCl, pH 6.8, containing 4% SDS, 10% mercaptoethanol, 20% glycerol, and 0.004% bromophenol blue) and heated at 95°C for 10 min. Proteins were separated by 10% SDS-PAGE for phosphoenolpyruvate carboxylase (PEPC), Rubisco, and glycine decarboxylase complex (GDC). The proteins were then transferred to a nitrocellulose membrane and immunoblotted (*n* = 3) with primary antibodies, PEPC and GDC H-subunits (1:1,000), purchased from PhytoLab (catalog numbers PHY0048 and PHY0655S). The band intensities were quantified with ImageJ 1.52 software (NIH, United States). Band intensities of the control treatments were assumed to be 100%, and upregulation and downregulation were expressed accordingly after using β-actin protein as the standard to calculate proteins in each sample.

### Analyses of Limitation in CO_2_ Assimilation and Stomatal Morphometry

Separation of the quantitative limitation analysis approach at saturating light was used. Disentangling the limitations of the net photosynthesis due to stomata (*L*_S_) and non-stomata (*L*_SN_) was estimated by the following expressions:

(9) $$L_{\text{S}}=\frac{P_{\text{n}}^{{}^{\prime \prime \prime }}-P_{\text{n}}^{{}^{\prime \prime }}}{P_{\text{n}}^{{}^{\prime \prime \prime }}}*100$$

(10) $$L_{\text{SN}}=\frac{P_{\text{n}}^{{}^{\prime \prime }}-P_{\text{n}}^{\prime }}{P_{\text{n}}^{{}^{\prime \prime \prime }}}*100$$

where $P_{\text{n}}^{\prime }$ is the net photosynthesis rate at ambient conditions in the greenhouse, $P_{\text{n}}^{{}^{\prime \prime }}$ is the *P*_n_ at *C*_atm_ = 400 μmol CO_2_ m^–2^ s^–1^, while $P_{\text{n}}^{{}^{\prime \prime \prime }}$ is the *P*_n_ at *C*_i_ = *C*_atm_ = 400 μmol CO_2_ m^–2^ s^–1^ (Farquhar and Sharkey, 1982; Bellasio et al., 2018).

Stomatal morphometric analyses were conducted with the peel-off method. Transparent nail polish was used to prepare slides (*n* = 3). The number of stomata, area of the guard cells (stomatal area), the roundness of the stomata, and the area of the stomatal aperture were estimated in the given area by using ImageJ 1.52 software (NIH, United States).

### Carbon Isotope Discrimination and Bundle Sheath Leakiness

The leaves used for photosynthesis measurements (third and fourth from the top) were dried and ground in a ball mill (MM200, Retch, Germany). The triplicate of 2 mg samples was packed into a tin capsule for ^13^C isotope determination using a mass spectrometer (IRMS IsoPrime 100 Elementar, a vario PYRO cube, Elementar, Germany). The following formula was used to calculate δ^13^C (Kubásek et al., 2007):

(11) $$\delta^{13}\,\text{C}=\left(\frac{R_{\text{p}}}{R_{\text{s}}}-1\right)*1,000$$

where *R*_p_ is the ^13^C/^12^C obtained from a mass spectrometer in plant samples and *R*_s_ is a reference value of ^13^C/^12^C in standard V-PDB (Vienna Pee Dee Belemnite); all values were expressed as per mil of dry weight. Carbon isotope discrimination (Δ^13^C) in the leaves was calculated from plant δ^13^C values (δ_p_) and air δ^13^C values (δ_a_) with the following formula (Farquhar et al., 1989):

(12) $${\text{Δ}}^{13}\,\text{C}=\frac{\delta_{\text{a}-}\,\delta_{\text{p}}}{1+\frac{\delta_{\text{p}}}{1,000}}$$

Bundle sheath leakiness (*φ*) was estimated according to Farquhar (1983) with the following expression:

(13) $$\varphi =\frac{\left[{\text{Δ}}^{13}\,\text{C}-a+\frac{\left(a-b_{4}\right)\,C_{\text{i}}}{C_{\text{atm}}}\right]}{\left[\frac{\left(b_{3}-s\right)\,C_{\text{i}}}{C_{\text{atm}}}\right]}$$

where *a*, *b*_3_, *b*_4_, and *s* are isotopic discrimination constants; *a* (4.4‰) is CO_2_ in air diffusivity through the stomata, *b*_3_ (29‰) is the carboxylation of Rubisco, *b*_4_ (−5.7‰) is the HCO_3_^–^ dissolution and fractionation of phosphoenolpyruvate (PEP) carboxylation, and *s* (1.8‰) is the leakage of CO_2_ from the bundle sheath to mesophyll cells.

### Statistical Analyses

The experiment was repeated twice in similar conditions of greenhouse and the data presented here as the mean ± SE (*n* = 3) for all parameters. Two-way analysis of variance (ANOVA) was performed to study differences in treatments, while a *post hoc* test (Bonferroni) was also calculated to examine the significant difference (*P* < 0.05) among the means of each treatment. Linear regressions were carried out between *P*_n_ vs. (*L*_S_, *L*_NS_, Δ^13^C, and *φ*), Δ^13^C vs. (ψ_w_, *C*_i_/*C*_a__tm_, WUEi, and *φ*), *φ* vs. (ψ_w_, *C*_i_/*C*_a__tm_, and WUEi), *g*_s_ vs. (*L*_S_ and WUEi), *L*_S_ vs. WUEi, *V*_cmax_ vs. *L*_NS_, and ΦPS2 vs. Φ_CO__2_ at various PPFDs in all treatments. These are shown as Supplementary Material.

## Results

### Plant Biomass and Water Relations

**FIGURE 1:**
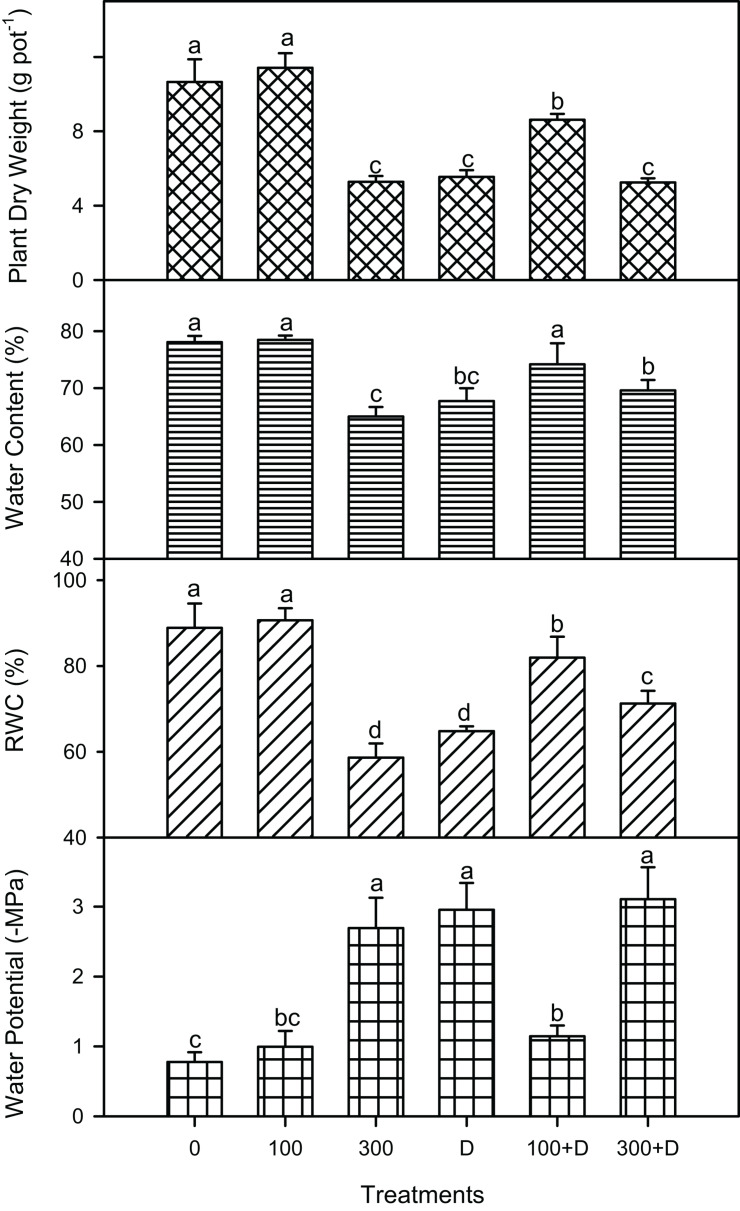
| Plant biomass and water relations under salinity, drought, and a combination of both treatments. Mean ± SE (*n* = 3) of plant dry weight (in grams per pot), leaf water content (percent of fresh weight), relative water content (*RWC*, in percent), and leaf water potential (in megapascals) for treatments of 0, 100, and 300 mM NaCl, drought (D), and a combination of salinity and drought [100 and 300 mM NaCl + drought (100 + D and 300 + D, respectively)]. *Different letters* denote, after Bonferroni *post hoc* test, significant difference (*P* < 0.05).

Salinity, drought, and a combination of both (S + D) affected the biomass of *P. antidotale* significantly (*P* < 0.001; Figure 1). Plant biomass did not only decline under individual treatments of high salinity and drought but also at 300 + D in comparison to the control treatment. Interestingly, combined treatment (100 + D, *P* < 0.001) led to a higher growth (significant differences in various parameters: dry weight, water content, and relative water content) than did drought alone. The water potential (negative values in megapascals) of leaves also showed a similar trend, with the exception of the 100 + D treatment, where it was similar to the control and 100 mM NaCl-treated plants.

### H_2_O_2_/CO_2_ Gas Exchange Parameters at Saturated Light

The *P*_n_ declined under provided stress treatments of either salt and drought alone or a combination of both (*P* < 0.001; Table 1). The maximum *P*_n_ was recorded under 100 mM NaCl treatment (statistically insignificant when compared to the control) and the lowest value was observed under 300 + D treatment. High salinity severely decreased the values of *P*_n_ when added to drought conditions, whereas low salinity enhanced the values when combined with drought (i.e., 100 + D). The other parameters of gaseous exchange, *g*_s_, *C*_i_, and *E* are also well correlated with *P*_n_ and with each other. The light response curves revealed CO_2_ saturation of the control and low-salinity-treated plants at a PPFD of 2,000 μmol photon m^–2^ s^–1^ while that of drought (alone)-treated plants at irradiance below 600 μmol photon m^–2^ s^–1^. The addition of salinity (both moderate and high) to drought conditions improved the saturation irradiance of the plants compared to that in response to drought alone (see *I*_s_ data; Table 1). The CO_2_ compensation point (*I*_c_), the quantum efficiency of CO_2_ (Φ_CO__2_), and dark respiration were also significantly affected by the applied treatments, while ETR/*P*_n__gross_ increased only in 300 mM NaCl. The highest intrinsic water use efficiency (WUEi) was recorded for drought alone (D) and 300 + D-treated plants.

**TABLE 1:** CO_2_/H_2_O gas exchange responses under salinity, drought, and the combination of both.

Gas exchange	Treatments					
	
	0	100	300	D	100 + D	300 + D
*P*_n_ (μmol m^–2^ s^–1^)	22.71*a*0.82	23.19*a*0.83	12.78*c*0.31	10.64*c*0.49	17.61*b*0.52	8.20*d*0.20
*g*_s_ (mol m^–2^ s^–1^)	0.149*a*0.012	0.122*b*0.010	0.074*d*0.003	0.044*e*0.003	0.109*c*0.009	0.039*e*0.001
*C*_i_ (μmol m^–2^ s^–1^)	235*a*18.43	175*b*6.03	107*d*4.76	98*e*6.65	151*c*3.76	62*f*4.22
*E* (mol m^–2^ s^–1^)	3.71*a*0.059	2.78*b*0.014	1.50*c*0.053	1.29*d*0.017	2.81*b*0.008	0.78*e*0.012
WUEi (*P*_n_*/g*_s_)	155*bc*18	193*b*18	171*bc*5	245*a*22	164*c*16	212*a*7
ETR/*P*_ngross_ (unitless)	4.61*b*0.21	4.78*b*0.06	6.79*a*0.81	4.67*b*0.07	4.45*b*0.13	4.28*b*0.13
*C*_i_/*C*_atm_ (unitless)	0.59*a*0.046	0.44*b*0.015	0.27*d*0.012	0.49*b*0.017	0.38*c*0.009	0.16*e*0.011
*R*_d_ (μmol m^–2^ s^–1^)	−0.65*b*0.07	−0.61*b*0.01	−0.36*a*0.06	−0.92*c*0.08	−1.76*e*0.08	−1.46*d*0.05
*I*_S_ (μmol m^–2^ s^–1^)	2,050*a*100	2,015*a*44	1,296*c*55	542*d*47	1,809*b*100	1,020*c*77
*I*_C_ (μmol m^–2^ s^–1^)	56.65*a*3.22	47.68*b*2.23	37.26*c*5.22	20.71*d*6.23	49.74*b*5.23	35.15*c*3.23
Φ_CO__2_ (mol CO_2_ μmol^–1^)	0.017*a*0.000	0.018*a*0.000	0.012*c*0.001	0.009*d*0.001	0.014*b*0.000	0.008*d*0.000
*V*_cmax_ (μmol m^–2^ s^–1^)	72.68*b*5.93	78.49*a*7.67	36.86*d*3.93	24.20*e*2.31	57.88*c*5.35	14.84*f*1.91
*J*_max_ (μmol m^–2^ s^–1^)	72.51*b*8.24	78.94*a*8.68	36.03*d*4.91	24.49*e*3.91	57.89*c*2.00	15.16*f*1.03

*The values of net photosynthesis (P_n_), stomatal conductance (g_s_), intercellular CO_2_ (C_i_), transpiration rate (E), water use efficiency (WUEi), ETR/P_ngross_, C_i_-to-CO_2_ in air ratio (C_i_/C_a_), dark respiration (R_d_), saturation light (I_s_), CO_2_ compensation point (I_c_), apparent quantum yield of CO_2_ assimilation (Φ_CO__2_), Rubisco carboxylase activity (V_cmax_), and RuBP regeneration (J_max_) are the mean of three replicates ± SE. Different letters express differences between treatments after Bonferroni post hoc test (P < 0.05). 0, 100, and 300 are millimolar concentrations of NaCl treatments, D is drought, and 100 + D and 300 + D are combinations of drought with 100 and 300 mM NaCl, respectively.*

**FIGURE 2:**
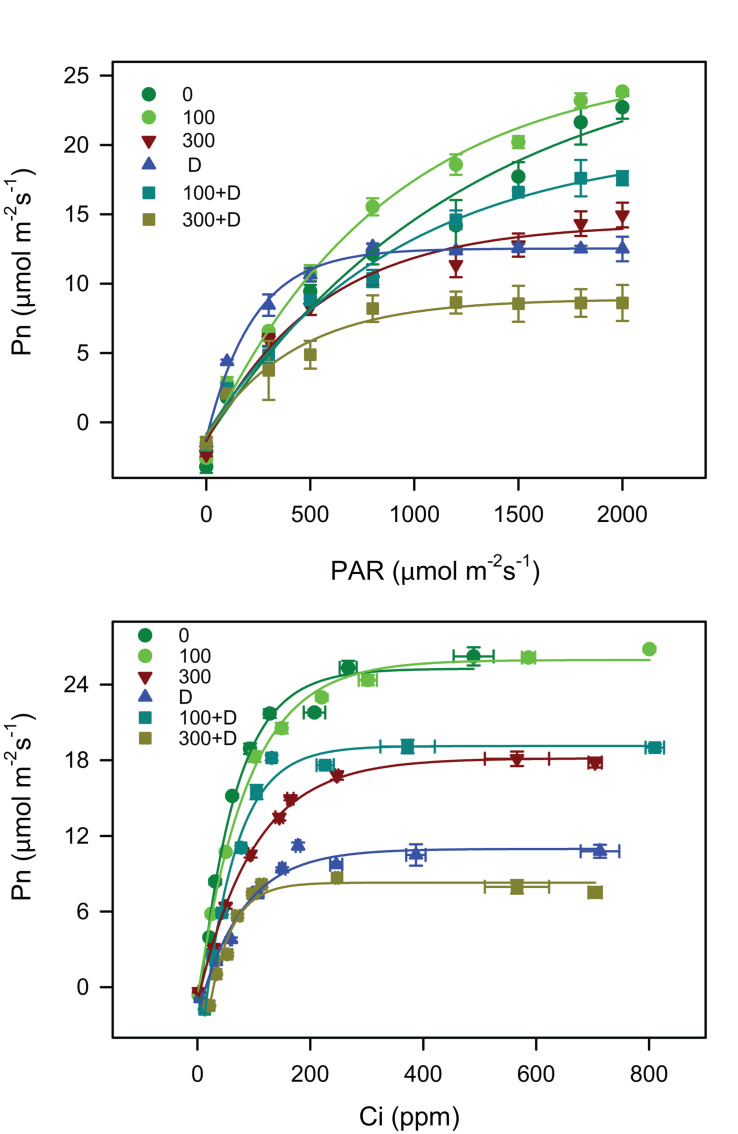
Light response curve (*P*_n_–PAR) and carbon response curve (*P*_n_n–*C*_i_) measurements under salinity, drought, and a combination of both treatments. *P*_n_–PAR curve represents the response of net photosynthesis in the range of 0–2000 μmol m^−2^ s^−1^ photosynthetic active radiation (PAR) and *P*_n_–*C*_i_ curve indicates the capacity of net photosynthesis under various intercellular CO_2_ concentrations (in micromoles per square meter per second) when CO_2_ in the air (*C*_atm_) is in the range of 0–1,500 μmol m^−2^ s^−1^. Values shown are the mean ± SE (*n* = 3) under all treatments, as described in Figure 1.

The CO_2_ response curves (Figure 2) were used to determine various photosynthetic parameters (Table 1). The *V*_cmax_ and *J*_max_ were unaffected at 100 mM NaCl, but significantly reduced in response to other treatments (300 mM NaCl, D, 100 + D, and 300 + D) when compared to the control. The combination of drought and low salinity enhanced *V*_cmax_ and *J*_max_ about 50% in comparison to drought only, but these values were still lower than 100 mM NaCl. The highest reduction was observed with the 300 + D treatment, where these parameters were about one-fifth of the values of the control.

**FIGURE 3:**
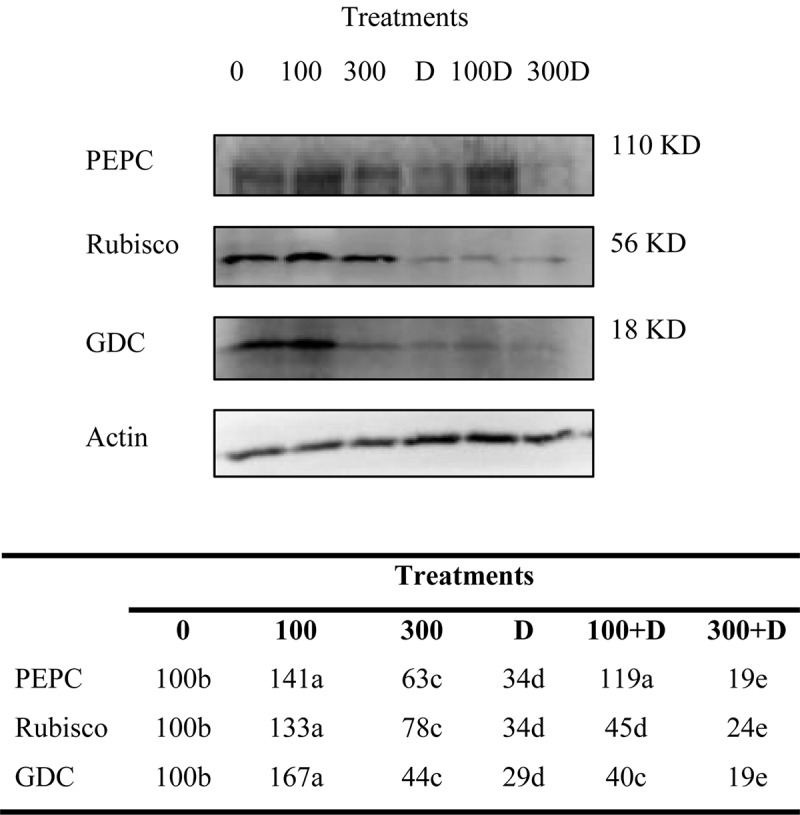
Variations of photosynthetic protein expressions. Immunoblots of phosphoenolpyruvate carboxylase (PEPC), ribulose 1-5, bisphosphate carboxylase/oxygenase (Rubisco), and glycine decarboxylase (GDC) expressed changes in the protein expressions under treatments of 0, 100, and 300 mM NaCl, drought (D), and a combination of salinity with drought [100 and 300 mM NaCl + drought (100 + D and 300 + D respectively)]. *Different letters* denote, after Bonferroni *post hoc* test, significant difference (*P* < 0.05).

Immunoblots of PEPC, Rubisco, and GDC contents in leaf tissues revealed significant changes under the treatments provided (Figure 3). The protein content increased in response to 100 mM NaCl, but decreased at 300 mM NaCl. A combination of high salinity and drought caused a severe reduction in their contents as compared to salt and drought alone.

### Carbon Isotope Discrimination and Stomatal and Non-stomatal Limitations During Photosynthesis

**FIGURE 4:**
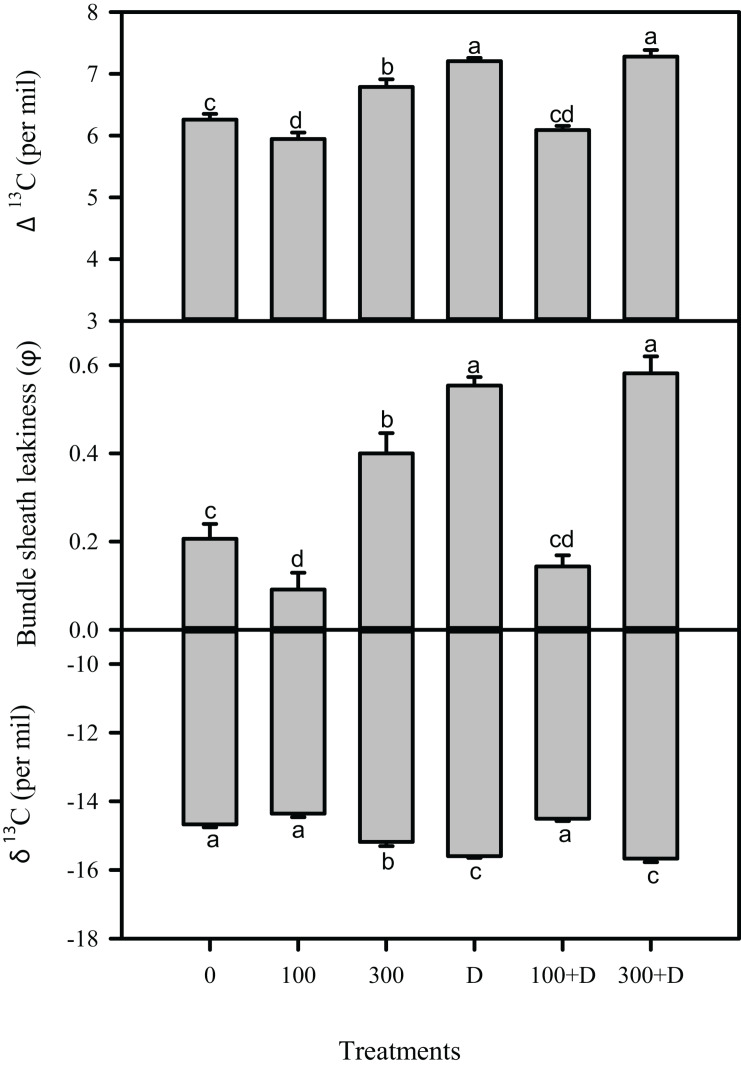
Measurement of carbon isotope in the leaf of *Panicum antidotale* under salinity, drought, and a combination of both treatments. Carbon isotope discrimination in the leaf (Δ^13^C), bundle sheath leakiness (*φ*), and carbon isotope in leaf (δ^13^C) showed variations under treatments of 0, 100, and 300 mM NaCl, drought (D), and a combination of salinity with drought [100 and 300 mM NaCl + drought (100 + D and 300 + D, respectively)]. *Different letters* denote, after Bonferroni *post hoc* test, significant difference (*P* < 0.05).

**FIGURE 5:**
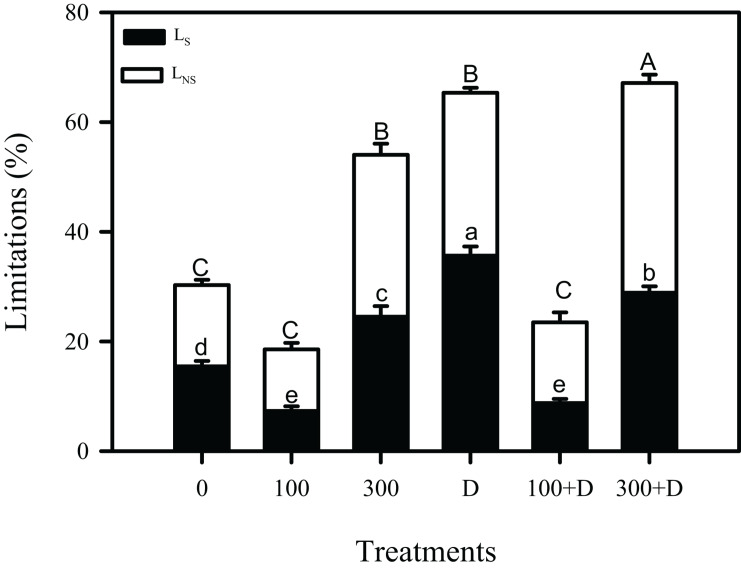
Contribution of limitation in photosynthesis estimation. *Dark* and *white bars* represent stomatal limitation (*L*_S_) and non-stomatal limitation (*L*_NS_), respectively, in the leaf of *Panicum antidotale* under treatments of 0, 100, and 300 mM NaCl, drought (D), and a combination of salinity with drought [100 and 300 mM NaCl + drought (100 + D and 300 + D, respectively)]. *Different letters* denote, after Bonferroni *post hoc* test, significant difference (*P* < 0.05).

Carbon isotope (δ^13^C) in leaf tissues was significantly reduced under high salinity, and a further reduction was recorded at drought and 300 + D treatments, in contrast to control plants. A similar trend was observed in leaf ^13^C isotope discrimination (Δ^13^C) among all treatments, while bundle sheath leakiness (*φ*) of CO_2_ was the highest in the 300 + D treatment (Figure 4). Figure 5 shows the quantitative contribution of photosynthetic limitations in response to the applied treatments. Stomatal limitation (*L*_S_) contributed about 25–30% at salinity (300 mM NaCl) and combination of salinity and drought (300 + D), while *L*_S_ was significantly higher at drought treatment (about 35%). The non-stomatal limitation (*L*_NS_) appears to be a major limiting factor of photosynthesis in response to the combination of salinity and drought (300 + D), although it accounted for at least 30% of limitation at 300 mM NaCl and drought treatment as well.

**FIGURE 6:**
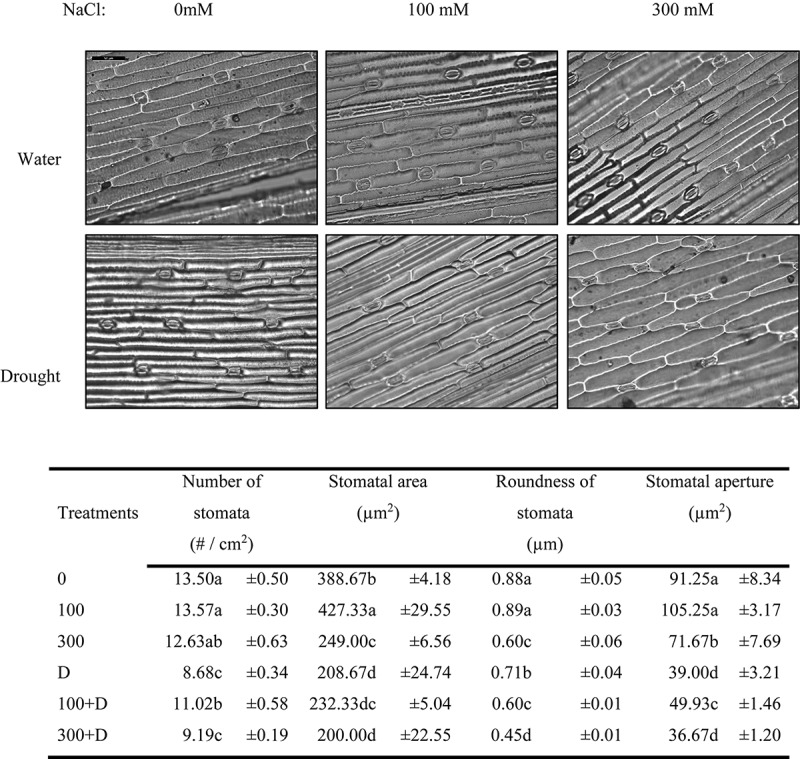
Stomatal morphometric characteristics of *Panicum antidotale*. Changes in the number of stomata, stomatal area, roundness of stomata, and area of the stomatal aperture for treatments of 0, 100, and 300 mM NaCl, drought (D), and a combination of salinity with drought [100 and 300 mM NaCl + drought (100 + D and 300 + D, respectively)] are shown. *Different letters* denote, after Bonferroni *post hoc* test, significant difference (*P* < 0.05).

The stomatal morphometric estimation showed a decreasing trend of stomatal area and aperture in salt, drought, and the combination of both, whereas the number of stomata was mainly decreased with the introduction of the drought condition. The 100 + D treatment showed increased values of these parameters as compared to drought alone (Figure 6).

### Chlorophyll *a* Fluorescence Parameters and Chlorophyll (SPAD)

**FIGURE 7:**
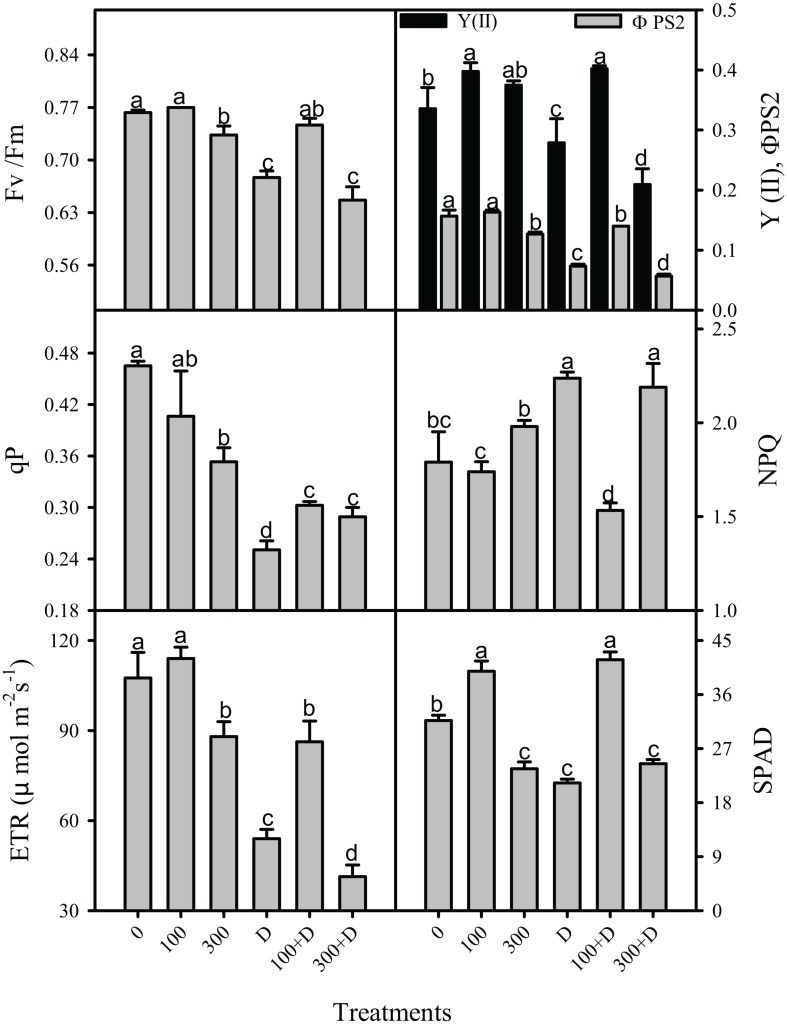
Responses of chlorophyll *a* fluorescence in the leaf of *Panicum antidotale* under salinity, drought, and a combination of both treatments. The mean ± SE (*n* = 3), maximum quantum efficiency of PS2 (*F*_v_/*F*_m_), apparent quantum efficiency of PS2 (ΦPS2, YII), photochemical quenching (qP), non-photochemical quenching (NPQ), electron transport rate (ETR), and chlorophyll (SPAD) for treatments of 0, 100, and 300 mM NaCl, drought (D), and a combination of salinity with drought [100 and 300 mM NaCl + drought (100 + D and 300 + D, respectively)] are shown. *Different letters* denote, after Bonferroni *post hoc* test, significant difference (*P* < 0.05).

Chlorophyll *a* fluorescence was measured simultaneously with the gas exchange parameters on the similar leaf in all the aforementioned treatments (Figure 7). The maximum and apparent quantum efficiency of PSII (*F*_v_/*F*_m_ and ΦPS2) remained unaffected under saline treatment (both low and high salinity), but with about 15 and 10% reductions in *F*_v_/*F*_m_, and 3.9- and 4.3-fold reductions in ΦPS2 were recorded under drought and 300 + D treatments, respectively, when compared to the control. These parameters were a little improved in response to not only 100 mM NaCl but also to 100 + D. In comparison to the control, photochemical quenching (qP) and ETR were reduced at salinity, drought, and the combination of both. The reduction in qP were about 31, 85, and 60% at 300 mM NaCl, drought, and 300 + D, respectively, while the reduction in ETR was more pronounced at the 300 + D treatment. The magnitude of heat dissipation (NPQ, non-photochemical quenching) increased by 20% at drought and 300 + D treatments when compared to the control. These aforementioned parameters of chlorophyll fluorescence had a better response at 100 + D when compared to drought treatment alone. In addition, SPAD values representing the chlorophyll content in the leaves also reveal a similar pattern, i.e., reduced content at high salinity, drought, and a combination of both.

### Correlation Between Various Parameters

The linear regressions were carried out between various parameters at salt, drought, and a combination of both treatments (Supplementary Material). The *r*^2^-values showed a strong correlation under all the treatments tested. The decline in net photosynthesis was well correlated (*P* < 0.001) with the various parameters (Supplementary Figure S1), and similarly the water potential was significantly correlated (*P* < 0.01) with the carbon isotope data (Supplementary Figure S1). WUEi was also correlated with *L*_s_ (*P* < 0.001, *r*^2^ = 0.51), *g*_s_ (*P* = 0.07, *r*^2^ = 0.60), and *φ* (*P* = 0.1, *r*^2^ = 0.48). The *r*^2^-values were low (∼0.2–0.3) with Δ^13^C and *φ*, while a high correlation was observed between Δ^13^C and *φ* (*P* < 0.001, *r*^2^ = 0.99). The reduction in stomatal conductance (*g*_s_) was well correlated (*r*^2^ = 0.71) with stomatal limitation of photosynthesis.

## Discussion

Salinity and water deficit conditions often coexist in nature, particularly in arid and semi-arid areas of the world. The purpose of this study was to determine the individual and combined effects of these stressors, with special emphasis on the photosynthesis performance of *Panicum antidotale*. A combined stress of low salinity and drought (100 + D) benefited the plants. However, high salinity combined with drought (300 + D) negatively affected several physiological processes.

### Synergetic Relationship Between Salinity and Water Deficit Condition

The synergetic effects of salinity and drought were observed at both 100 + D and 300 + D treatments (Figure 1). Similar effects have been reported for the halophyte *Zygophyllum xanthoxylum* (Ma et al., 2012) and some cultivars of barley (Ahmed et al., 2013). Although the plant growth was not stimulated by low salinity, as reported for other monocotyledon halophytes (Flowers and Colmer, 2008; Koyro et al., 2013; Hussain et al., 2015), a combination of low salinity and drought (100 + D treatment) enhanced plant biomass in contrast to drought treatment alone. Thus, the role of the uptake of Na^+^ and Cl^–^ ions, i.e., cheap osmotica, in reducing the drastic effects of water deficit conditions can be presumed. This assumption is further evidenced by the high values of the relative water content (RWC) and water content (WC) in this treatment (Figure 1). On the other hand, high salinity combined with drought (300 + D) did not cause any further decrease in plant biomass when compared to both (high salinity and drought) treatments alone. This response is antagonistic to that recorded at the 100 + D treatment, i.e., reduction in biomass.

### Photosynthetic Adaptation Under Salinity, Drought, and Combined Stress Conditions

Measurements of CO_2_/H_2_O in our test species clearly indicated the different responses under single (salinity or drought) and combined stresses (Table 1). Low salinity coupled with drought (100 + D) buffered the negative impact of drought on gaseous exchange. However, alone (100 mM NaCl), it did not cause any significant increase in net photosynthesis. We, therefore, assume that the hazardous impacts of drought conditions on photosynthetic performance can be lessened by combining drought stress and moderate salinity. This can be explained as enhanced activities of certain enzymes of the C_4_ cycle by chloride ions (Guo et al., 2018). Analyses of the PEP carboxylase expression also validated this hypothesis. The accumulated ions contribute to osmotic adjustment (Flowers and Colmer, 2008) by allowing plants to uptake water (i.e., RWC) when very little water is available in the medium. The uptake of water in such a stressful environment facilitates stomatal conductance (*g*_s_), which ensures CO_2_ influx to the photosynthesizing cells (Flowers and Colmer, 2008; Ma et al., 2012; Hussain et al., 2015).

A further increase in salinity led to an antagonistic plant response. The plants could not cope with the combined stress treatment (300 + D), probably because of the excessive intake of ions, as an effort to adjust osmotically, causing ion toxicity or imbalance. Thus, the closure of the stomata (low *g*_s_ and stomatal aperture) (Table 1 and Figure 6), as a strategy to conserve plant water, resulted in lower values of intercellular CO_2_ (*C*_i_) and C fixation, as explained in Ashraf (2003) and Asrar et al. (2017). Net photosynthesis and gaseous exchange were significantly decreased in these plants compared to high salinity and drought alone. On the contrary, *C*_i_ remained unchanged in a number of C_4_ species under drought or salinity (Koyro et al., 2013; Asrar et al., 2017). This variation may be due to the different experimental setup, non-stomatal limitations such as CO_2_ leakage (see below), species specificity, and/or different tolerance limits under various stresses.

C_4_ plants are equipped with a competitively better photosynthetic machinery, i.e., the presence of PEP carboxylase in mesophyll cells. This enzyme is highly efficient in fixing carbon even when the concentration of *C*_i_ becomes low due to stomatal closure (Way et al., 2014). Thus, the plants are benefited with minimal loss of water and improved photosynthesis as compared to C_3_ plants. Our results clearly indicate that the stomatal limitation (*L*_S_) in *Panicum* is an adaptive strategy to conserve water by reducing transpirational rates and improving WUEi (Table 1), particularly in response to drought and 300 + D treatments. This may be an advantageous effect, but at the cost of low carbon assimilation, similar to other C_4_ plants (Ashraf, 2003; Galmés et al., 2017).

Diffusional resistance or leakiness of CO_2_ into bundle sheath cells affects photosynthesis in drought- and/or salinity-treated C_4_ plants (Farquhar and Sharkey, 1982; Farquhar, 1983; Ellsworth and Cousins, 2016). An enhanced leakage of CO_2_, in drought treatment and combined stress (300 + D), decreased the efficiency of the C_4_ carbon-concentrating mechanism, which is evidenced by the reduced expression of PEP carboxylase (Figure 3). Such findings have been previously discussed (Cernusak et al., 2013; Tomás et al., 2013; Kromdijk et al., 2014). In contrast to the control (no salinity and no drought) plants, low values of bundle sheath leakiness (*φ*) and a high expression of PEP carboxylase in plants treated with 100 mM NaCl and combined stress (100 + D) demonstrate the beneficial effects of these treatments on the C_4_ cycle, probably due to the intact leaf anatomy (e.g., Kranz anatomy). The high values of carbon isotopes in these plants represent no restriction to the carboxylation activity of Rubisco, hence the higher net photosynthesis and less photorespiration (Leisner et al., 2010; Moinuddin et al., 2017). Besides, regression analyses showed an inverse correlation of Δ^13^C with *P*_n_ (*r*^2^ = 0.87, *P* < 0.01; Supplementary Figure S1). Hence, we conclude that moderate salinity (100 mM NaCl) improves C_4_ photosynthesis and alleviates the negative impact of drought (Leisner et al., 2010; Moinuddin et al., 2017). However, the reduced content of Rubisco at 100 + D limited photosynthesis in these plants. The negative impact of high salinity and drought treatments on the content of Rubisco was discussed earlier by Koyro et al. (2013), and we recorded a severe reduction in its content by combining both stresses (Figure 3). The content of carbon isotopes is considered a reliable indicator of the water use efficiency in C_4_ plants (Cernusak et al., 2013; Caemmerer et al., 2014; Kromdijk et al., 2014; Ellsworth and Cousins, 2016). Accordingly, the regression analysis illustrated a direct relationship (*r*^2^ = 0.48, *P* = 0.05) between Δ^13^C and WUEi in this study.

Biochemical processes, in addition to stomatal limitations, also affect CO_2_ assimilation (Von Caemmerer, 2000; Long and Bernacchi, 2003). An enhanced carboxylation rate of Rubisco (*V*_cmax_), the continuous supply of RuBP (i.e., higher *J*_max_), and carbon utilization for sucrose and starch synthesis (Φ_CO__2_) at 100 mM NaCl kept the photosynthetic rates comparable to plants of the control (i.e., no stress) treatment (Table 1). Combining moderate salinity with drought (100 + D) caused a slight decrease in these biochemical processes. On the other hand, the stressful effects of high salinity and drought treatments were visible, with significant reductions of *V*_cmax_, *J*_max_, and Φ_CO__2_, which led to a decline in *P*_n_ and plant biomass. These biochemical limitations were even more pronounced when the plants were exposed to combined stresses, i.e., 300 + D, indicating impairment in the light-independent phase of photosynthesis. The reason behind such a finding may be either the downregulated activity of enzymes of the Calvin cycle or the reduced supply of products of the light-dependent phase (i.e., ATP and NADPH). Also, the toxic effects of accumulated ions such as Na^+^ and Cl^–^ on photosynthesis cannot be neglected (Pérez-López et al., 2012; Asrar et al., 2017; Galmés et al., 2017). These assertions are further strengthened by the expression analysis of a large subunit of Rubisco (Figure 3) and the regression analysis between *P*_n_ and non-stomatal limitation (*L*_NS_) (*r*^2^ = 0.93, *P* < 0.001) (Supplementary Figure S1). The photosynthetic performance of *Panicum* has been limited due to stomata (*L*_S_) (Ashraf, 2003) and other biochemical limitations (Koyro et al., 2013) under salinity, and it has been confirmed by the estimation of the contributions of *L*_NS_ and *L*_S_ (Figure 5). Nevertheless, more limiting factors of photosynthesis (such as light and mesophyll conductance) need to unravel from a set of complicated calculations, particularly for C_4_ plants. Nevertheless, photochemical reactions of photosynthesis are discussed (see below) by measurement of the chlorophyll fluorescence.

### Light-Harvesting and Chlorophyll Variations Under Salinity, Drought, and Combined Stress Conditions

The electron transfer during light reactions of photosynthesis provides energy for the synthesis of NADPH and ATP. These products are vital for the carbon assimilation reactions. Chlorophyll fluorescence and chlorophyll content were used as non-invasive indicators to determine impairment (if any) in light reactions of photosynthesis (Genty et al., 1989; Baker, 2008). The comparatively low values of *F*_v_/*F*_m_ (the maximal photochemical efficiency) in response to drought, high salinity, and a combination of both (Figure 7) indicate severe damage to the PSII reaction centers (Farquhar et al., 1989; Duarte et al., 2013; Chen et al., 2016), which is further evidenced by the decrease in the actual efficiency of photosystem II, i.e., Y(II) and ΦPSII. Similar findings exist for several Mediterranean species under water deficit conditions (Oxborough and Baker, 1997; Bellot et al., 2004; Flexas et al., 2012a). The reduction in the electron transport rate of the plants treated with drought, 300 mM NaCl, and 300 + D corresponded to their low stomatal conductance and suggested a reduction in light-harvesting capacity. It prevents the otherwise inevitable photo-oxidative stress, excess generation of reactive oxygen species (ROS), and membrane lipid peroxidation (Dalal and Tripathy, 2018). This assumption is further supported by the decreased contents of photosynthetic pigments (e.g., chlorophyll). Thus, the capacity to fix light energy decreases (Manaa et al., 2019) and plants are light-saturated at low values of irradiance (*I*s; Table 1). The findings of this study indicate a strict check and balance between *P*_n_ and the light-harvesting and photoprotection capacity of *Panicum*.

Non-photochemical quenching (NPQ) serves as a photoprotective mechanism to prevent photo-oxidative stress in plants (Külheim et al., 2002). The observed increase in NPQ in response to drought, high salinity, and a combination of both (300 + D) demonstrates the dissipation of excessive energy *via* the xanthophyll cycle. Such a regulation of unutilized light energy in light reactions minimizes the chance of injury to the thylakoid membrane that can, otherwise, be caused by overly produced ROS, particularly when the biochemical and photochemical reactions are disrupted (Bellot et al., 2004; Koyro et al., 2013; Manaa et al., 2019). The low risk of ROS (e.g., O_2_^–^, ^–^OH, and H_2_O_2_) toxicity is also reflected by the adjusted ETR (as discussed above) and the low ETR/*P*_ngross_ ratio in plants. Further, the low expression of GDC under high salinity, drought, and their combination (300 + D), reveals an unregulated carbon flow between photosynthesis and photorespiration, which may also lead to developing a balance between respiratory metabolites (e.g., glycine) and increased cellular mole ratios of CO_2_/O_2_ (Timm et al., 2018; Giuliani et al., 2019). In contrast, *Panicum* treated with low salinity (alone and in combination with drought) efficiently regulated photochemical reactions, i.e., Y(II), ΦPSII, qP, ETR, and, therefore, net photosynthesis.

## Conclusion

This study demonstrates that an addition of 100 mM NaCl to dry soil (100 + D) minimized the deleterious effects of water deficit on the biomass and photosynthetic performance of *P. antidotale*. This is due to the positive effects of low-salinity treatment on the photochemical reactions by stimulating photosynthetic pigments and PSII efficiency (YII) to harvest light energy for the efficient synthesis of ATP and NADPH. Increases in stomatal area, enzymes involved in bioenergetics (PEP carboxylase, Rubisco, and glycine carboxylase), and the biochemical reactions [i.e., the carboxylation rate of Rubisco (*V*_cmax_) and RuBP regeneration (*J*_max_)] contributed to an overall higher CO_2_ assimilation in these plants. Combining low salinity with drought (100 + D) lowered the CO_2_ leakage, enriching CO_2_ in the bundle sheath cells to allow maximum carboxylase activities of Rubisco. However, it was the reduction in the content of Rubisco that limited the photosynthetic performance in these plants (100 + D). In contrast, the combination of high salinity (300 mM NaCl) and drought had a severe impact on the photosynthetic performance (*P*_n_) than both the stresses alone. Although high salinity lessened the damaging effect of drought on stomatal limitations (number of stomata and stomatal conductance) photochemical (PSII efficiency, ETR) and biochemical (*V*_cmax_ and *J*_max_) reactions, non-stomatal limitations, mainly the metabolic enzymes (PEP carboxylase, Rubisco, and glycine carboxylase), were significantly enhanced in the combination of these stresses (300 + D). The risk of photo-oxidative stress in these plants was lowered by limiting the light-harvesting capacity. Plant biomass (i.e., FW) was even improved due to better management of the plant water status (RWC and WUEi), demonstrating a beneficial effect of the osmotic component at such a high salinity.

The shown comparative study of the individual and combined effects of salinity and drought conditions on *Panicum* opens new doors for a better understanding of plant responses in their natural environment, where multiple environmental constraints are present simultaneously. Nevertheless, the type of soil in different habitats of plant may vary and, therefore, the impact of soil properties on setting particular stress conditions must be kept into account while analyzing large fields.

## Data Availability Statement

The datasets generated for this study are available on request to the corresponding author.

## Author Contributions

TH and XJL conceived the idea. TH executed the experiments and drafted the manuscript. XTL helped out in conducting immunoblot experiments. H-WK reviewed the manuscript critically and checked the calculations. WZ contributed to setting up the experiment. BG improved the manuscript and idea.

## Conflict of Interest

The authors declare that the research was conducted in the absence of any commercial or financial relationships that could be construed as a potential conflict of interest.
