# Supplementary material for: Low Salinity Improves Photosynthetic Performance in Panicum antidotale Under Drought Stress
Source: Front Plant Sci. 2020 May 29;11:481. doi: 10.3389/fpls.2020.00481 (PMC7273886; doi:10.3389/fpls.2020.00481)
Supplement: Supplementary file 1 [file Table_1.docx]

**Table. Supplementary**. Two way ANOVA representing the *F* and *P* values under drought and salinity treatments dry weight, leaf WC, RWC (%), water potential, net photosynthesis (Pn), stomatal conductance (gs), intercellular CO_2_ (Ci), transpiration rate (E), water use efficiency (WUEi), Ci to CO_2_ in air ratio (Ci/Catm), saturation light (Is), CO_2_ compensation point (Ic), Rubisco carboxylase activity (V_cmax_), RuBP regeneration (J_max_), dark respiration (Rd), Chlorophyll (SPAD), maximum quantum efficiency of PS2 (Fv/Fm), actual quantum efficiency of PS2 (YII), quantum efficiency of PS2 (ɸPS2), apparent quantum yield of CO_2_ assimilation (ɸ_CO2_), photochemical quenching (qP), non-photochemical quenching (NPQ), electron transport rate (ETR), stomatal limitation (L_S_), non-stomatal limitation (L_NS_), carbon isotopes discrimination (Δ ^13^C), bundle sheath leakiness (φ), and carbon isotopes (δ ^13^C).

|  | **Salinity** | | **Drought** | | **Salinity x Drought** | |
| --- | --- | --- | --- | --- | --- | --- |
| **Parameters** | ***F*** | ***P*** | ***F*** | ***P*** | ***F*** | ***P*** |
| Dry weight | 40.12 | < 0.0001 | 14.24 | 0.003 | 9.02 | 0.004 |
| Leaf WC | 46.63 | 0.001 | 0.00 | 0.978 | 7.09 | 0.009 |
| RWC (%) | 60.20 | 0.001 | 17.81 | 0.001 | 44.51 | < 0.0001 |
| Water potential | 322.47 | 0.001 | 239.82 | 0.001 | 116.31 | < 0.0001 |
| Pn | 147.60 | 0.001 | 242.95 | 0.001 | 24.28 | < 0.0001 |
| gs | 32.67 | 0.001 | 71.15 | 0.001 | 20.77 | < 0.0001 |
| Ci | 116.50 | 0.001 | 28.13 | 0.001 | 0.30 | 0.75 |
| E | 561.39 | 0.001 | 598.51 | 0.001 | 252.20 | 0.001 |
| WUEi | 0.95 | 0.413 | 6.91 | 0.022 | 7.10 | 0.009 |
| Ci/Catm | 116.50 | 0.001 | 28.13 | < 0.0001 | 0.30 | 0.75 |
| Is | 46.50 | 0.001 | 118.26 | < 0.0001 | 31.72 | 0.001 |
| Ic | 11.84 | 0.001 | 60.53 | < 0.0001 | 32.56 | 0.001 |
| V_cmax_ | 823.23 | < 0.0001 | 1269.39 | < 0.0001 | 113.07 | 0.001 |
| J_max_ | 632.36 | < 0.0001 | 928.30 | < 0.0001 | 84.03 | 0.001 |
| Rd | 22.50 | < 0.0001 | 278.16 | < 0.0001 | 32.25 | 0.001 |
| SPAD | 131.09 | < 0.0001 | 7.73 | 0.017 | 18.55 | 0.001 |
| Fv/Fm | 22.61 | < 0.0001 | 61.10 | 0.001 | 6.60 | 0.012 |
| Y(II) | 85.70 | < 0.0001 | 46.77 | 0.001 | 32.37 | < 0.0001 |
| ɸPS2 | 256.47 | < 0.0001 | 728.91 | 0.001 | 73.98 | < 0.0001 |
| φCO_2_ | 114.45 | < 0.0001 | 178.32 | 0.001 | 21.67 | < 0.0001 |
| qP | 21.39 | < 0.0001 | 484.07 | 0.001 | 49.44 | < 0.0001 |
| NPQ | 21.55 | < 0.0001 | 5.63 | 0.035 | 9.26 | 0.004 |
| ETR | 59.45 | < 0.0001 | 257.08 | < 0.0001 | 8.41 | 0.005 |
| L_S_ | 131.56 | < 0.0001 | 67.65 | < 0.0001 | 30.55 | 0.001 |
| L_NS_ | 102.11 | < 0.0001 | 57.06 | < 0.0001 | 7.55 | 0.008 |
| Δ ^13^C | 61.55 | < 0.0001 | 47.17 | < 0.0001 | 9.13 | 0.004 |
| φ | 61.55 | < 0.0001 | 47.17 | < 0.0001 | 9.13 | 0.004 |
| δ ^13^C | 61.56 | < 0.0001 | 47.14 | < 0.0001 | 9.12 | 0.004 |

The confidence level was selected to 95%.
