# Supplementary material for: Low Salinity Improves Photosynthetic Performance in Panicum antidotale Under Drought Stress
Source: Front Plant Sci. 2020 May 29;11:481. doi: 10.3389/fpls.2020.00481 (PMC7273886; doi:10.3389/fpls.2020.00481)
Supplement: Supplementary file 2 [file Image_1.pdf]

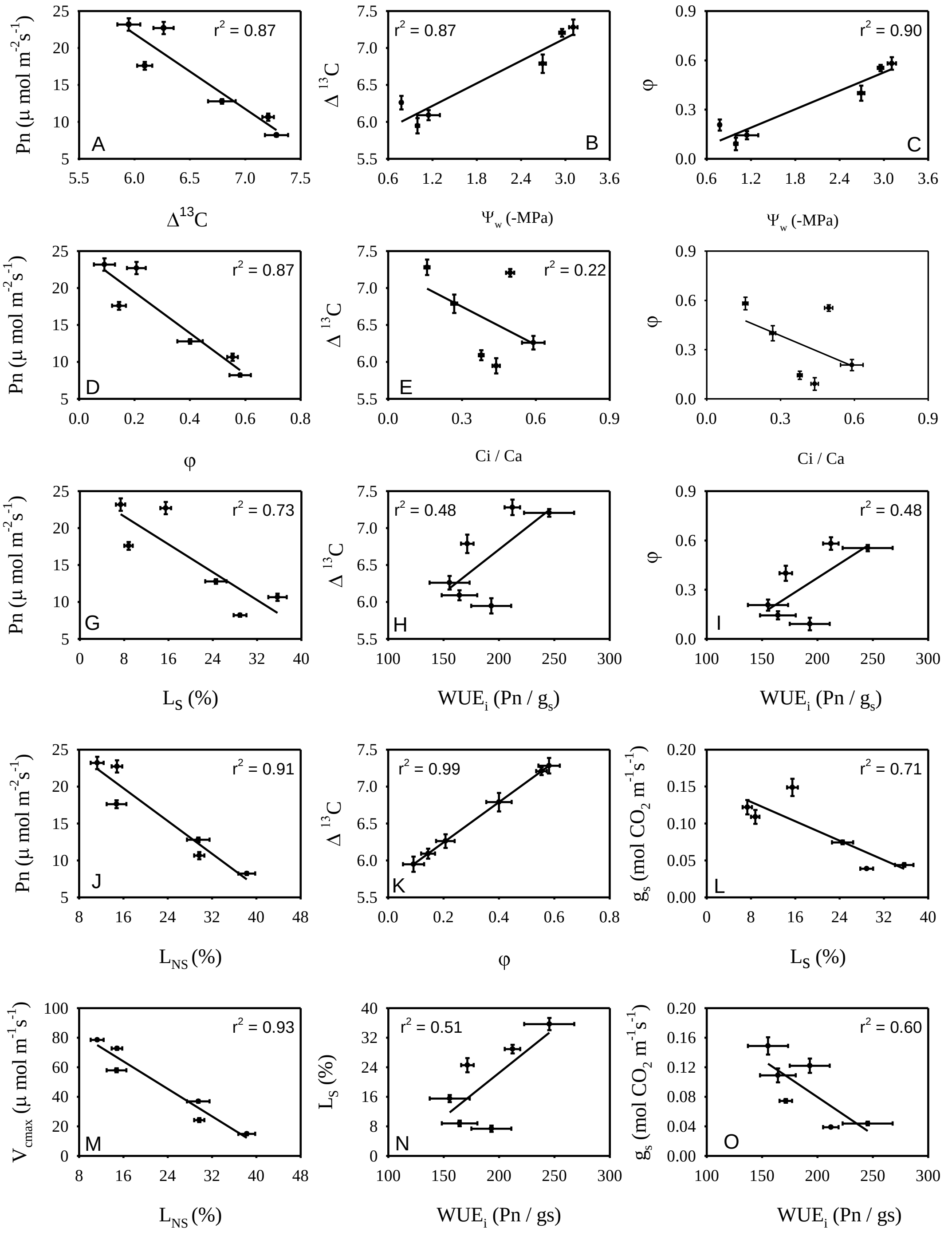

Fig S1. Supplementary. Linear regressions estimated the relationship between various parameters tested. Relationship between net photosynthesis (Pn) and (A) Carbon isotope discrimination **(**$\Delta\ ^{13}C$), (D) bundle sheath leakiness (φ), (G) stomatal limitation (L_S) and (J) non-stomatal limitation (L_{NS}), relationship between Carbon isotope discrimination **(**$\Delta\ ^{13}C$) and (B) water potential (ψ_w), (E) Ci/Catm , (H) water use efficiency (WUEi) and (K) bundle sheath leakiness (φ), relationship between bundle sheath leakiness (φ) and (C) water potential (ψ_w), (F) Ci/Catm, (I) water use efficiency (WUEi), relationship between stomatal conduct (gs) and (L) stomatal limitation (L_S) and (O) non-stomatal limitation (L_{NS}), relationship between maximum carboxylation capacity of Rubisco (V_{cmax}) and (M) non-stomatal limitation (L_{NS}), and relationship between stomatal limitation (L_S) and (N) water use efficiency (WUEi).
